# Prognostic Value of Radiological Response to Chemotherapy in Patients with Osteosarcoma

**DOI:** 10.1371/journal.pone.0070015

**Published:** 2013-07-29

**Authors:** Shinji Miwa, Akihiko Takeuchi, Toshiharu Shirai, Junichi Taki, Norio Yamamoto, Hideji Nishida, Katsuhiro Hayashi, Yoshikazu Tanzawa, Hiroaki Kimura, Kentaro Igarashi, Akishi Ooi, Hiroyuki Tsuchiya

**Affiliations:** 1 Department of Orthopaedic Surgery, Kanazawa University School of Medicine, Kanazawa, Japan; 2 Department of Biotracer Medicine, Kanazawa University School of Medicine, Kanazawa, Japan; 3 Department of Molecular and Cellular Pathology, Kanazawa University School of Medicine, Kanazawa, Japan; University Hospital of Navarra, Spain

## Abstract

**Background:**

Chemotherapy is essential to improve the prognosis of the patients with osteosarcoma, and the response to chemotherapy is an important prognostic factor. In this study, the impact of various radiological examinations on overall survival (OS) and event-free survival (EFS) was evaluated.

**Method:**

Eighty-two patients with high-grade osteosarcoma were included in this study, and we evaluated the following factors for prognostic significance: age (≥40 years), gender (male), tumor location (truncal site), metastatic disease, histological response to chemotherapy, radiological response to chemotherapy assessed using X-ray, angiography, CT, MRI, ^201^Tl scintigraphy, and ^99m^Tc-MIBI scintigraphy (^99m^Tc-MIBI), and combined radiological score (CRS).

**Results:**

Univariate analyses revealed that metastatic disease, histological response, ^99m^Tc-MIBI, and CRS were significantly correlated with OS. Multivariate analyses showed that metastatic disease (OS: HR 35.9, *P*<0.001; EFS: HR 17.32, *P*<0.001) was an independent predictor of OS and EFS. Tumor location (HR 36.1, *P* = 0.003), histological response (HR 31.1, *P* = 0.036), and ^99m^Tc-MIBI (HR 18.4, *P* = 0.038) were significant prognostic factors for OS. Moreover, CRS was a marginally significant predictor of OS and EFS.

**Conclusion:**

The chemotherapeutic effects evaluated by ^99m^Tc-MIBI and CRS could be considered as prognostic factors in osteosarcoma.

## Introduction

Osteosarcoma is the most common primary malignant bone tumor and primarily affects adolescents. Multidisciplinary approaches, including chemotherapy and wide resection surgery, dramatically improve the treatment outcomes for patients with osteosarcoma; however, metastatic lesions and poor response to chemotherapy have been reported as important prognostic factors as well [1], [2]. The response to chemotherapy is commonly assessed by histological analysis, although it is difficult to evaluate the chemotherapeutic effects before tumor excision. On the other hand, we previously reported that Tc-99m-methoxyisobutyl-isonitrile scintigraphy (^99m^Tc-MIBI) and combined radiological score (CRS) revealed significant correlation with histological response to chemotherapy [3], [4]. Radiological examination is useful to evaluate the chemotherapeutic effects before surgical treatment. At our institute, patients with osteosarcoma are examined using X-ray photography (X-ray), angiography, magnetic resonance imaging (MRI), Thallium-201 scintigraphy (^201^Tl), and ^99m^Tc-MIBI before chemotherapy as well as after 3–5 courses of chemotherapy. Although there are many reports regarding the prognostic significance of histological response to chemotherapy, there are few reports that have assessed the prognostic value of radiological examinations in the treatment of osteosarcoma. Thus, we investigated the correlation between response to chemotherapy and clinical outcomes in patients with osteosarcoma.

## Methods

### Patients

Between May 1992 and July 2012, 137 patients with osteosarcoma were treated at Kanazawa University Hospital. The patients received X-ray, computed tomography (CT), MRI, and bone scintigraphy for the diagnosis and assessment of clinical staging. All the tumors were confirmed pathologically from the specimens obtained from biopsy and surgery. Among the 137 patients, 82 with clinical records, who were diagnosed as high-grade osteosarcoma and who received chemotherapy and surgical treatment, were included in the present study (Table 1). Patients with low-grade osteosarcoma and those who did not undergo surgery or receive chemotherapy were excluded from this study. This study was approved by the Institutional Review Board of the Kanazawa University Graduate School of Medical Science, Kanazawa, Japan. Written informed consent was obtained from all patients and/or their family. All the patients received 3–5 courses of preoperative chemotherapy at intervals of 2–3 weeks [5]. In each chemotherapy course, cisplatin (120 mg/m^2^) was continuously infused through a catheter for 1–2 h followed by 48 h of continuous doxorubicin infusion (30 mg/m^2^/day for 2 days) and 72 h of caffeine infusion (1.5 g/m^2^/day for 3 days). Before chemotherapy and after 3–5 courses of chemotherapy, the chemotherapeutic effects were evaluated using X-ray, angiography, MRI,^ 201^Tl, and ^99m^Tc-MIBI. Three to five courses of chemotherapy were administered, and subsequently, surgery was performed. All the tumors were surgically resected, and the chemotherapeutic effects were evaluated histologically. Further, 5 or 6 courses of additional chemotherapy were administered using ifosfamide (3 g/m^2^/day for 3 days), etoposide (60 mg/m^2^/day for 3 days), and caffeine (1.5 g/m^2^/day for 3 days). Follow-up evaluation to detect local recurrence and metastasis consisted of X-ray, CT, MRI, bone scan, and ^201^Tl scan.

**Table 1:** Characteristics of the study patients.

Characteristic	No.
Follow up period (months)	49.5 (6–190)
Age at diagnosis (y)	22.1 (5–69)
<40	68
≥40	14
Gender	
Male	48
Female	34
Primary tumor location	
Femur	48
Tibia	21
Humerus	5
Pelvis	4
Other	4
Pathologic subtype	
Osteoblastic	58
Chondroblastic	16
Fibroblastic	4
Other	4
Surgical approach	
Limb salvage	78
Amputation	4
Surgical stage	
IIA	2
IIB	52
IIIB	28
Huvos grade	
I (<50% necrosis)	8
II (50–89% necrosis)	24
III (≥90% necrosis)	20
IIIB (100% necrosis)	30

### Image Analyses

All the 82 patients were assessed using X-ray, 64 using angiography, 77 using MRI, 68 using ^201^Tl, and 57 using ^99m^Tc-MIBI before chemotherapy and after 3–5 courses of chemotherapy. The images were assessed by radiologists without any knowledge of the histological response and clinical outcomes of these patients.

X-ray images were assessed on the basis of cortical recovery and continuity, and angiograms were assessed on the basis of changes in tumor vascularity (Table 2). On the basis of these results, patients were classified into the following groups: complete response (CR), partial response (PR), stable disease (SD), or progressive disease (PD). CR and PR were classified as responders.

**Table 2:** Radiological evaluation method for chemotherapeutic effect.

	Definition of the responders	Point
X-ray	Sclerotic change and/or cortical remodeling	1
Angiogram	Disappearance of pathologic vascularization	1
MRI	ΔEM ≥30%	2
^201^Tl	ΔUR ≥30%	2
^99m^Tc-MIBI	ΔUR ≥30%	2
Combined radiological score	Total point ≥60%	

ΔEM: the percentage reduction in extraskeletal masses (EM).

ΔUR: the percentage reduction in uptake ratio (UR).

Combined radiological score (CRS) = Total of the radiological score/Full marks (%).

MR images were assessed on the basis of reduction of the maximum diameter of the extraskeletal mass (EM) [6]. The percentage reduction in EM (ΔEM) was calculated as follows:
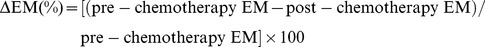



Both ^99m^Tc-MIBI and ^201^Tl images were assessed by the reduction in uptake ratio (UR) [3], [4]. For quantitative analysis of the images, a manual region of interest (ROI) was set on the tumor site, and a symmetrical ROI was set on the contralateral normal area as a background. UR in ^99m^Tc-MIBI and ^201^Tl were calculated by dividing the count density of the lesion by that of the background ROI. For assessment of the chemotherapeutic effect, the percentage reduction in uptake ratio (ΔUR) was defined as follows:
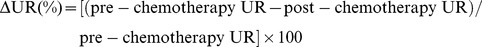



On the basis of the images, patients who demonstrated ΔEM ≥30% in MRI, ΔUR ≥30% in ^201^Tl, and ΔUR ≥30% in ^99m^Tc-MIBI were classified as responders [3], [4], [6].

We recently reported a combined radiological scoring system, which revealed a strong correlation with the histological response to chemotherapy (Table 2) [4]. For patients who were classified as good responders by X-ray or angiography, 1 extra point was added to the total number of points. For patients who were classified as good responders by MRI, ^201^Tl, or ^99m^Tc-MIBI, 2 extra points were added to the total number of points. Combined radiological scores (CRS) were defined as follows:
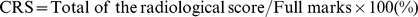



For patients who could not be assessed using some of the imaging methods, the combined radiological score was calculated from the obtained images. Patients who demonstrated CRS ≥60% were classified as responders [4].

### Histological Evaluation of Chemotherapeutic Effects

Resected specimens were assessed according to histological response to chemotherapy. In patients with tumor-bearing bones that were reused as autografts, specimens of the resected bone were histologically assessed. Histological response to chemotherapy was assessed according to the degree of cellularity and necrosis in the largest slice of the resected tumor [7]. Grade IV (100% necrosis) and grade III (90%–99% necrosis) were considered as good responses to chemotherapy, whereas grade II (50%–89% necrosis) and grade I (0%–49% necrosis) were considered as poor responses to chemotherapy.

### Statistical Analysis

The factors of patient age (<40 and ≥40 years), gender, tumor location (extremity or trunk), metastatic disease, histological response (<90% and ≥90% necrosis), and radiological response (X-ray, angiography, MRI, ^201^Tl, ^99m^Tc-MIBI, and CRS) were subjected to univariate analyses. Overall survival (OS) and event-free survival (EFS) were calculated using the Kaplan–Meier method along with the log rank test. In addition, the Cox proportional hazards regression model was used for multivariate analyses to identify independent predictors of OS and EFS. Any factor with *P*<0.4 in the univariate analysis was included in the multivariate Cox proportional hazards model. OS was defined as the time from the initial diagnosis to death from any cause. On the other hand, EFS was defined as the time from the initial diagnosis to metastasis, local recurrence, or death from any cause. Statistical significance was defined as *P*<0.05. Marginal statistical significance was defined as 0.05≤*P*<0.10. Statistical analyses were performed using the EZR statistical software (Saitama Medical Center, Jichi Medical University, Saitama, Japan).

## Results

### Patients

A total of 82 patients with high-grade osteosarcoma, [48 male and 34 female; mean age, 22.1 (range, 5–69) years], who received chemotherapy and surgical treatment and could be reviewed through clinical records, were retrospectively enrolled in this study (Table 1). The mean follow-up period was 49.5 months (range, 6–190 months). At initial diagnosis, 28 patients (34.1%) demonstrated clinically detectable metastases, and 54 patients (65.9%) did not present any metastasis. Histologically, 58 patients were classified as osteoblastic type, 16 patients as chondroblastic type, 4 as fibrous type, and 4 as “other” type. The tumors were located in the femur in 48 cases, tibia in 21, humerus in 5, pelvis in 4, fibula in 2, clavicle in 1, and calcaneus in 1. On the basis of the Enneking’s surgical staging, 2 patients were classified as stage IIA, 52 as stage IIB, and 28 as stage IIIB [8].

**Figure 1:**
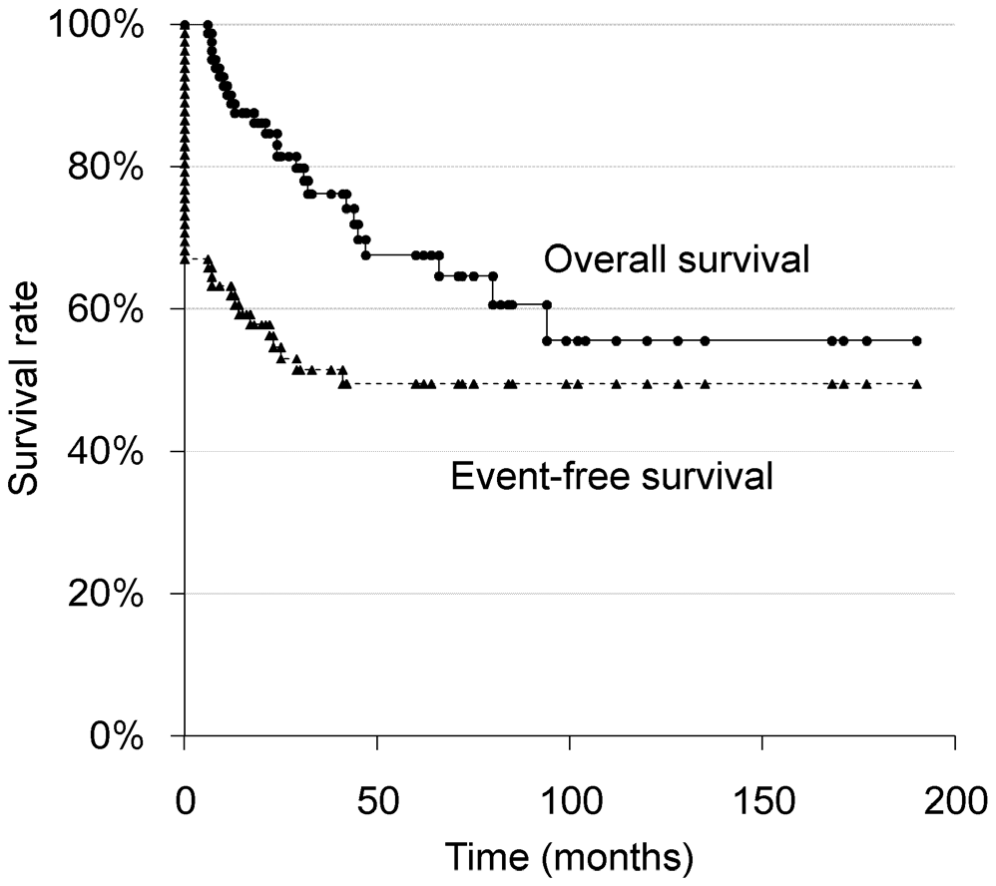
Overall and event-free survival of all 82 patients.

The 3-, 5-, 10-year OS and EFS rates of the 82 patients were 76.2, 67.6, and 55.6%, and 51.4, 49.6, and 49.6%, respectively (Fig. 1).

### Overall Survival (OS)

**Figure 2:**
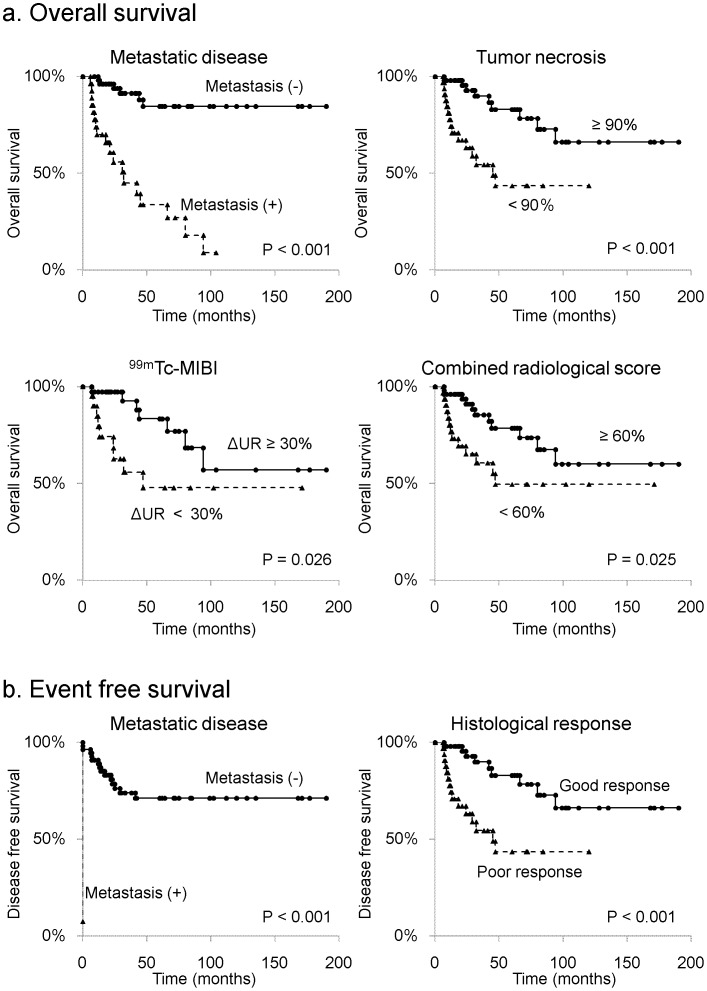
Kaplan-Meier curves of overall survival and event-free survival for variables with prognostic significance in univariate analyses. a. Overall survival. b. Event-free survival.

Kaplan–Meier analysis demonstrated that metastases at initial diagnosis (*P*<0.001), histological response (*P*<0.001), ^99m^Tc-MIBI response (*P* = 0.026), and CRS (*P* = 0.025) were significantly associated with OS (Table 3). The 5-year OS rate of the patients with metastases at initial diagnosis was 33.8%, whereas the 5-year OS rate of the patients without metastasis was 84.6% (Fig. 2a). In addition, the 5-year OS rate of the patients with <90% necrosis was 43.6%, whereas the 5-year OS rate of the patients with ≥90% was 83.0% (Fig. 2a). Moreover, the 5-year OS rate of the patients who presented <30% ΔUR in ^99m^Tc-MIBI was 47.8%, whereas the 5-year OS rate of the patients who presented ≥30%ΔUR in ^99m^Tc-MIBI was 83.4% (Fig. 2a). Furthermore, the 5-year OS rate of the patients who revealed <60% CRS was 49.6%, whereas the 5-year OS rate of the patients revealed ≥60% CRS was 78.6% (Fig. 2a).

**Table 3:** Univariate analysis of overall survival.

Charasteristic		5-year OS	*P*
Age	<40 years	69.0	0.732
	≥40 years	63.3	
Gender	Male	76.6	0.098
	Female	52.3	
Location	Extremity	70.3	0.312
	Trunk	40.0	
Metastasis	Absent	84.6	<0.001**
	Present	33.8	
Histological response	necrosis<90%	83.0	<0.001**
	necrosis≥90%	43.6	
X-ray	Good	70.9	0.691
	Poor	58.4	
Angiography	Good	74.7	0.051
	Poor	39.1	
MRI	ΔEM≥30%	76.7	0.113
	ΔEM<30%	58.4	
^201^Tl	ΔUR≥30%	74.5	0.209
	ΔUR<30%	63.6	
^99m^Tc-MIBI	ΔUR≥30%	83.4	0.026*
	ΔUR<30%	47.8	
CRS	≥60%	78.6	0.025*
	<60%	49.6	

* *P*<0.05,

** *P*<0.01.

Multivariate analysis revealed that metastatic disease at initial diagnosis [hazard ratio (HR) = 35.9; *P*<0.001], truncal site (HR = 36.1; *P* = 0.003), histological response (HR = 31.1; *P* = 0.036), and ^99m^Tc-MIBI response (HR = 18.4; *P* = 0.038) were significant independent predictive factors of OS (Table 4). CRS was a marginally significant predictive factor of OS (HR = 141.5; *P* = 0.059).

**Table 4:** Multivariate Cox models of overall survival for study patients.

Charasteristic	HR	95% CI	*P*
Male	0.58	0.16–2.13	0.408
Location (truncal site)	36.05	3.46–375.20	0.003**
Metastatic disease	35.88	5.77–223.30	<0.001**
Histological response	31.13	1.25–773.00	0.036*
Angiography	2.36	0.52–10.62	0.264
MRI	2.13	0.26–17.18	0.479
^201^Tl	2.35	0.22–25.32	0.482
^99m^Tc-MIBI	18.42	1.18–286.90	0.038*
CRS	141.54	0.83–24102	0.059

* *P*<0.05,

** *P*<0.01.

### Event-free Survival (EFS)

Kaplan–Meier analysis demonstrated that metastasis at initial diagnosis (*P*<0.001) and histological response (*P* = 0.040) were significantly associated with EFS (Table 5). The 5-year EFS of the patients with metastases was 0%, whereas the 5-year EFS rate of the patients without metastasis was 71.2% (Fig. 2b). In addition, the 5-year EFS of the patients with <90% necrosis was 33.7%, whereas the 5-year EFS rate of the patients with ≥90% necrosis was 60.7% (Fig. 2b).

**Table 5:** Univariate analysis of event-free survival.

Charasteristic		5-year EFS	*P*
Age	<40 years	49.1	0.517
	≥40 years	53.9	
Gender	Male	52.6	0.350
	Female	45.7	
Location	Extremity	49.8	0.904
	Trunk	44.4	
Metastasis	Absent	71.2	<0.001**
	Present	0	
Histological response	necrosis<90%	60.7	0.040*
	necrosis≥90%	33.7	
X-ray	Good	47.2	0.478
	Poor	57.6	
Angiography	Good	55.6	0.143
	Poor	33.3	
MRI	ΔEM≥30%	52.6	0.501
	ΔEM<30%	44.7	
^201^Tl	ΔUR≥30%	54.9	0.565
	ΔUR<30%	47.0	
^99m^Tc-MIBI	ΔUR≥30%	55.8	0.385
	ΔUR<30%	43.3	
CRS	≥60%	54.5	0.328
	<60%	42.0	

* *P*<0.05,

** *P*<0.01.

Multivariate analysis revealed that only metastatic disease at initial diagnosis was a significant independent predictor of EFS (HR = 17.3; *P*<0.001; Table 6). Moreover, CRS was a marginally significant predictor of EFS (HR = 8.85; *P* = 0.079).

**Table 6:** Multivariate Cox models of event-free survival for study patients.

Charasteristic	HR	95% CI	*P*
Male	0.769	0.31–1.92	0.573
Metastatic disease	17.32	3.89–77.20	<0.001**
Histological response	3.13	0.62–15.78	0.166
Angiography	1.91	0.68–5.38	0.221
^99m^Tc-MIBI	3.89	0.74–20.51	0.110
CRS	8.85	0.78–100.58	0.079

** *P*<0.01.

## Discussion

There are a large number of reports regarding prognostic factors for patients with osteosarcoma such as age, truncal location, gender, metastatic lesions at initial diagnosis, chemotherapeutic effects, tumor size, alkaline phosphatase (ALP), recurrence, and P-glycoprotein [1], [2], [9]–[15]. Among these factors, histological analysis of excised tumors remains the most accurate method to assess the response to chemotherapy, and the significance of this method in the prognosis of patients with osteosarcoma is well documented [1], [2]. The present study also indicated that tumor necrosis was significantly correlated with OS, which is in accordance with the findings of previous reports [1], [2].

In the treatment of osteosarcoma, various radiological examinations, including X-ray, MRI, ^201^Tl, ^99m^Tc-MIBI, and positron emission tomography, are often performed before and after preoperative chemotherapy to assess extent of tumor invasiveness and cortical destruction and to plan the surgical treatment [3], [4], [6], [16]. Radiological examinations reflect the response to chemotherapy [3], [4], [6], and although X-ray is cheap and convenient, the results are often inaccurate. For patients who receive intra-arterial chemotherapy, angiography is also a convenient tool and depicts tumor vascularity, which reflects the chemotherapeutic effects. However, angiography is invasive for patients treated with intravenous chemotherapy, and the quantification of tumor blood flow using angiography is difficult. On the other hand, CT and MRI can be evaluated quantitatively and offer more accurate imaging of the local extent of osteosarcoma, which influences the surgical margins [6]. Reportedly, the changes in tumor size calculated using CT and MRI were significantly associated with the prognosis of the patients with osteosarcoma [6]. However, differentiation between residual tumors and fibrotic tissues using MRI is difficult, even with contrast enhancement [17]. In addition, the usefulness of ^201^Tl and ^99m^Tc-MIBI to assess the chemotherapeutic effects has been reported, and each of these can be used for quantitative assessment [3], [4], [18]–[24]. The accumulation of ^201^Tl depends on sodium–potassium pump activity and cellular chloride co-transport system and is useful for assessment of tumor activity and differentiation between residual tumors and fibrotic tissue [25]. On the other hand, ^99m^Tc-MIBI also accumulates in malignant tumors, and this accumulation depends on the mitochondrial function [26]–[28]. Assessment of the chemotherapeutic response by using only one radiological examination is limited in cases with no extraskeletal mass, few tumor vessels, and low accumulation of ^201^Tl and ^99m^Tc-MIBI. Recently, we developed a combined radiological scoring system, which revealed strong correlation with the histological response to chemotherapy [4].

Surgical margins can be reduced in patients whose radiological examinations indicate good response to chemotherapy. Intentional marginal excision has an outcome similar to that of wide excision in patients who show a complete response to chemotherapy [5]. On the other hand, a poor response to preoperative chemotherapy makes it difficult to reduce surgical margins and salvage the affected limbs. Although chemotherapeutic effects are important information to make the surgical treatment plan, it is impossible to assess chemotherapeutic effects before tumor excision. Therefore, preoperative radiological evaluation of the chemotherapeutic effects is extremely important and needs to be strongly correlated with the histological response and prognosis. We previously reported that ^99m^Tc-MIBI and CRS were strongly correlated with the histological responses to chemotherapy [3], [4]. In the present study, ^99m^Tc-MIBI revealed a significant association with OS in the patients with osteosarcoma. On the other hand, CRS revealed a marginally significant correlation with OS and EFS. Taken together, these findings suggest that ^99m^Tc-MIBI and CRS could be used to assess the chemotherapeutic effects.

### Conclusions

Tumor necrosis revealed significant correlation with OS as previously reported. In addition, ^99m^Tc-MIBI response and CRS correlate with histologic response and are now confirmed as prognostic factors in patients with high-grade osteosarcoma receiving preoperative chemotherapy and surgical resection.
